# Practical Pathology: A Manual for Students and Practitioners

**Published:** 1883-12

**Authors:** 


*Practical Pathology: A Manual for Students and Practitioners.* By G. Sims Woodhead, M.D., F.R.C.P.E. Edinburgh: Young J. Pentland. 1883.

There has long been felt by student and practitioner alike, the need of some work on Practical Pathology, which shall teach us how to get at the various organs of the body by post-mortem examination; what to observe when the cadaver is opened; and how to recognise the various lesions which have either directly caused, or have indirectly contributed to death. We then want a book to tell us concisely and clearly the various characteristics of disease as they are written on the organs, before section, and after it, on the cut surface. Then, how to harden and preserve pieces of these, how to cut them for microscopic examination, and lastly, how to stain, mount, and examine them.

Now, we consider that the work before us satisfies our demands. It opens by giving us full instructions as to the way of conducting a post-mortem examination; it then accurately describes the morbid appearances of the organs as seen on the outside, as well as on section of the whole organ. Then we are told how to preserve and harden pieces of the diseased tissue; the best stains to use; what we see under the low power of the microscope (50 diameters); and lastly, what the high power (300 diameters) reveals.

Now, this mode of investigation is systematic and thorough, and if properly carried out cannot fail to yield good results. It is the plan adopted in the University of Edinburgh, in which the author is at present Demonstrator of Practical Pathology, and conducts the class of students who are working at this subject under the superintendence of Professor Greenfield; and, under the management of Professors Sanders, Hamilton, and Greenfield, it has proved itself to be the only thorough way of teaching the subject.

Besides clear and practical description of morbid conditions, we find in this book another very excellent and striking feature, and that is the really beautiful coloured illustrations, of which there are nearly 150 given us. These illustrations are not only pretty to look at, but, seeing that they are mostly correct copies from nature, having been drawn largely from the author's own collection, and seeing that Dr. Woodhead has stained them with fluids which are most capable of showing up the diseased structure, and has coloured his plates in exact imitation of these stains, they are especially valuable and highly instructive.

Looking, then, at the immense amount of practical information which is contained in this book, the clearness of most of the descriptions of lesions, and the beauty and faithfulness of the numerous plates, we consider that Dr. Woodhead is to be congratulated on this his first effort as an author, and we hope that the book will have the large circulation that it deserves.

A special word of praise is due for the admirable manner in which the publisher and printer have carried out their work—far above what we are accustomed to in England, and quite equal to the best American publications.

